# Survival following segmentectomy or lobectomy in elderly patients with early-stage lung cancer

**DOI:** 10.18632/oncotarget.7704

**Published:** 2016-02-25

**Authors:** Yang Zhang, Chongze Yuan, Yawei Zhang, Yihua Sun, Haiquan Chen

**Affiliations:** ^1^ Department of Thoracic Surgery, Fudan University Shanghai Cancer Center, Shanghai, China; ^2^ Department of Oncology, Shanghai Medical College, Fudan University, Shanghai, China; ^3^ Shanghai Chest Hospital, Shanghai Jiao Tong University, Shanghai, China; ^4^ Institutes of Biomedical Sciences, Fudan University, Shanghai, China

**Keywords:** non-small cell lung cancer, lobectomy, segmentectomy

## Abstract

**Purpose:**

To determine the survival following segmentectomy versus lobectomy in elderly patients with early-stage non-small cell lung cancer (NSCLC).

**Methods:**

We identified 12324 elderly (≥ 70 years) patients with stage I ≤ 3 cm NSCLC in the Surveillance, Epidemiology and End Results (SEER) database. Propensity score methods were used to balance baseline characteristics of patients undergoing segmentectomy or lobectomy. Overall survival (OS) and lung cancer-specific survival (LCSS) of patients treated with segmentectomy versus lobectomy were compared in Cox regression models after adjusting, stratifying or matching patients based on propensity scores.

**Results:**

Cox models adjusting, stratifying or matching propensity scores all showed that patients treated with segmentectomy had significantly worse OS and LCSS compared to lobectomy. Subgroup analysis of patients with tumors ≤ 2cm, aged ≥ 75 years, or had ≥ 7 lymph nodes examined also revealed survival advantage associated with lobectomy.

**Conclusion:**

Elder age alone could not justify the application of segmentectomy in early-stage lung cancer. Prospective randomized trials are warranted to validate our results.

## INTRODUCTION

Controversy remains as to whether sublobar resection including segmentectomy and wedge resection could be a reasonable alternative to lobectomy in the treatment of early-stage non-small cell lung cancers (NSCLC). Compared to lobectomy, sublobar resection is considered to preserve pulmonary function, thus provide a better chance of surgical resection for a second primary lung cancer. The only randomized controlled trial (RCT) comparing lobectomy and sublobar resection in early-stage lung cancer (T1N0) showed that lobectomy was associated with a lower rate of local recurrence and a tendency towards better overall survival [1]. However, a high proportion (32.8%) of patients in the sublobar resection group in this RCT received wedge resection, while segmentectomy is considered to be a better choice of limited resection since its anatomical resection and more extensive lymph node dissection. A recent study comparing segmentectomy and wedge resection using the Surveillance, Epidemiology and End Results (SEER) database showed that segmentectomy was associated with significant improvement in overall and lung cancer-specific survival [2]. Moreover, elderly patients with lung cancer generally had higher postoperative morbidity and mortality rates, shorter life expectancy and worse pulmonary reserve. As a result, limited resection may be a more reasonable alternative to lobectomy in elderly patients with early-stage lung cancer. In this study, we used SEER database to compare the survival outcomes following segmentectomy versus lobectomy in elderly patients ≥ 70 years old with stage I NSCLC ≤ 3 cm in size.

## RESULTS

A total of 12324 patients were included in this study; 11503 received lobectomy and 821 underwent segmentectomy. Detailed patient characteristics were listed in Table 1. There were no significant differences regarding race, marital status, year of diagnosis, tumor histology or tumor grade between the two treatment groups. Patients undergoing segmentectomy were significantly older, were more likely to be females, had smaller tumor size, had less lymph nodes examined and were more likely to receive postoperative radiation therapy. Pretreatment baseline characteristics were balanced between the two resection groups after adjusting for propensity scores.

**Table 1:** Characteristics of elderly (≥ 70 years) patients with stage I ≤ 3 cm non-small cell lung cancer undergoing lobectomy or segmentectomy in SEER database, 1998-2012

Variables	Lobectomy (n = 11503)	Segmentectomy (n = 821)	*P*	
			Without Adjustment	With Adjustment
Age (years)				
70-74	5164 (44.9%)	309 (37.6%)	< 0.001	0.874
75-79	4053 (35.2%)	284 (34.6%)		
≥ 80	2286 (19.9%)	228 (27.8%)		
Mean±SD	75.8±4.3	76.7±4.8	< 0.001	0.987
Gender			0.025	0.920
Female	6263 (54.4%)	480 (58.5%)		
Male	5240 (45.6%)	341 (41.5%)		
Race			0.342	0.245
White	10189 (88.6%)	730 (88.9%)		
African American	590 (5.1%)	48 (5.8%)		
Others and unknown	724 (6.3%)	43 (5.2%)		
Marital status			0.177	0.230
Married	6495 (56.5%)	444 (54.1%)		
Unmarried	4656 (40.5%)	357 (43.5%)		
Unknown	352 (3.1%)	20 (2.4%)		
Year of diagnosis			0.071	0.505
1998-2004	4698 (40.8%)	309 (37.6%)		
2005-2012	6805 (59.2%)	512 (62.4%)		
Tumor size (mm)				
≤ 20	5783 (50.3%)	493 (60.0%)	< 0.001	0.840
21-30	5720 (49.7%)	328 (40.0%)		
Mean±SD	20.9±6.2	19.4±6.3	< 0.001	0.962
Histology			0.393	0.171
Adenocarcinoma	7296 (63.4%)	506 (61.6%)		
Squamous	3102 (27.0%)	224 (27.3%)		
Large cell	333 (2.9%)	30 (3.7%)		
Adenosquamous	327 (2.8%)	21 (2.6%)		
NOS	445 (3.9%)	40 (4.9%)		
Differentiation			0.229	0.450
Well	2069 (18.0%)	130 (15.8%)		
Moderate	5097 (44.3%)	350 (42.6%)		
Poor	3285 (28.6%)	260 (31.7%)		
Undifferentiated	216 (1.9%)	18 (2.2%)		
Unknown	836 (7.3%)	63 (7.7%)		
No. of LNs examined			< 0.001	NA
0	529 (4.6%)	250 (30.5%)		
1-6	4682 (40.7%)	393 (47.9%)		
7-17	4120 (35.8%)	87 (10.6%)		
18 or more	872 (7.6%)	18 (2.2%)		
Unknown	1300 (11.3%)	73 (8.9%)		
Radiation			< 0.001	NA
Yes	172 (1.5%)	28 (3.4%)		
No	11232 (97.6%)	789 (96.1%)		
Unknown	99 (0.9%)	4 (0.5%)		

Abbreviations: SD, standard deviation; NOS, not otherwise specified; LN, lymph nodes; NA, not adjusted.

Cox model including propensity score as a continuous variable showed that patients treated with segmentectomy had significantly worse overall survival (HR = 1.280, 95% CI: 1.158-1.414, *P* < 0.001) and lung cancer-specific survival (HR = 1.330, 95% CI: 1.158-1.528, *P* < 0.001) than those who underwent lobectomy (Table 2). When the survival analysis was conducted within strata defined by propensity score quintiles, we also found patients treated with lobectomy had better OS (HR ranged from 1.101-1.547) and LCSS (HR ranged from 1.129-1.647), although statistical significance was not reached in some categories. In the matched analysis, lobectomy also showed superiority regarding both OS (HR: 1.281, 95% CI: 1.102-1.489, *P* = 0.001) and LCSS (HR: 1.489, 95% CI: 1.199-1.849, *P* < 0.001).

**Table 2:** Comparison of survival following segmentectomy vs. lobectomy using propensity score analysis

Model	Overall survival		Lung cancer-specific survival	
	HR (95% CI)	*P*	HR (95% CI)	*P*
Adjusted for propensity scores	1.280 (1.158-1.414)	< 0.001	1.330 (1.158-1.528)	< 0.001
Propensity score quintiles				
1, lowest probability of segmentectomy	1.547 (1.197-1.999)	0.001	1.647 (1.174-2.311)	0.004
2	1.297 (1.022-1.646)	0.032	1.232 (0.884-1.718)	0.218
3	1.101 (0.869-1.394)	0.427	1.227 (0.892-1.687)	0.209
4	1.188 (0.955-1.478)	0.122	1.129 (0.826-1.543)	0.445
5, highest probability of segmentectomy	1.345 (1.109-1.631)	0.003	1.499 (1.138-1.974)	0.004
Matched analysis	1.281 (1.102-1.489)	0.001	1.489 (1.199-1.849)	< 0.001

Abbreviations: HR, hazard ratio; CI, confidence interval.

Subgroup analysis was first performed in patients with tumors ≤ 2 cm in size (Table 3). In the model adjusting for propensity scores, patients receiving lobectomy had a better OS (HR: 1.133, 95% CI: 0.983-1.306, *P* = 0.086) and LCSS (HR: 1.213, 95% CI: 0.993-1.483, *P* = 0.059) with borderline statistical significance. Similar results regarding OS (HR: 1.194, 95% CI: 0.972-1.467, *P* = 0.092) and LCSS (HR: 1.310, 95% CI: 0.970-1.769, *P* = 0.078) were found in the matched analysis. We then investigated patients ≥ 75 years old. Patients treated with lobectomy had significantly better OS and CSS in models adjusted for propensity scores (OS, HR = 1.239, 95% CI: 1.093-1.405, *P* = 0.001; LCSS, HR = 1.308, 95% CI: 1.094-1.563, *P* = 0.003) and matched analysis (OS, HR = 1.343, 95% CI: 1.117-1.613, *P* = 0.002; LCSS, HR = 1.443, 95% CI: 1.106-1.884, *P* = 0.007). Finally, we limited the survival analysis in patients who had 7 or more lymph nodes examined. In the Cox model adjusting for propensity scores, patients treated with lobectomy had a significantly better LCSS (HR = 1.493, 95% CI: 1.010-2.208, *P* = 0.044). HR also favored lobectomy in matched analysis, yet without statistical significance.

**Table 3:** Subgroup propensity score analysis comparing survival following segmentectomy vs. lobectomy

Subgroup	Overall survival		Lung cancer-specific survival	
	HR (95% CI)	*P*	HR (95% CI)	*P*
**Tumors ≤ 2 cm**				
Adjusted for propensity scores	1.133 (0.983-1.306)	0.086	1.213 (0.993-1.483)	0.059
Matched analysis (n = 982)	1.194 (0.972-1.467)	0.092	1.310 (0.970-1.769)	0.078
**Age ≥ 75 years**				
Adjusted for propensity scores	1.239 (1.093-1.405)	0.001	1.308 (1.094-1.563)	0.003
Matched analysis (n = 1018)	1.343 (1.117-1.613)	0.002	1.443 (1.106-1.884)	0.007
**Lymph nodes examined ≥ 7**				
Adjusted for propensity scores	1.112 (0.809-1.527)	0.514	1.493 (1.010-2.208)	0.044
Matched analysis (n = 210)	1.230 (0.762-1.988)	0.397	1.421 (0.769-2.625)	0.262

Abbreviations: HR, hazard ratio; CI, confidence interval.

## DISCUSSION

Elderly patients with lung cancer generally have inferior prognosis than their younger counterparts [4], and are usually under-represented in clinical trials [5, 6]. Several retrospective studies with relatively small numbers suggested that elderly patients with early-stage lung cancer might have similar survival outcomes following limited resection (including segmentectomy and wedge resection) compared to those receiving lobectomy [7–10]. Elderly patients usually had declined cardiopulmonary reserve and a limited life expectancy, thus supported the application of limited resections with reduced morbidity and lung function preservation. Kilic and colleagues [9] retrospectively investigated the oncological efficacy of lobectomy and segmentectomy in 184 (78 segmentectomy and 106 lobectomy) elderly (more than 75 years old) patients with stage I NSCLC. They found patients treated with segmentectomy or lobectomy had comparable disease-free survival and overall survival. However, this large population-based study showed that elderly patients ≥ 70 years undergoing segmentectomy had significantly worse OS and LCSS compared to those receiving lobectomy. Similar results were found in the subgroup analysis of patients ≥ 75 years old. Our results suggested that elder age alone could not justify the application of segmentectomy.

Oncological outcomes following segmentectomy remains controversial in stage I NSCLC ≤ 2 cm in size. Some retrospective studies and meta-analysis [11, 12] showed that survival outcomes following segmentectomy was inferior to lobectomy even in small-sized (≤ 2 cm) stage I NSCLC. However, the oncological efficacy was reported to be comparable among patients receiving the two resection types in several Japanese cohorts [13–15]. We found that lobectomy yielded a better OS and LCSS in elderly patients with stage I ≤ 2 cm NSCLC, yet with borderline statistical significance. The ongoing JCOG0802 trial [16] specifically comparing segmentectomy and lobectomy in ≤ 2 cm stage I NSCLC is expected to clarify this issue, although patients ≥ 70 years are still likely to be under-represented.

Number of lymph nodes examined is an important prognostic factor in node-negative NSCLC. Osarogiagbon and colleagues [17] analyzed pN0 NSCLC in the SEER database, and found a sequential decrease in mortality risk with increased number of lymph nodes examined, and the lowest mortality risk occurred in patients with 18-21 lymph nodes examined, while the median number of lymph nodes examined was only 6. In this study, we found that patients receiving segmentectomy had significantly less lymph nodes examined than those undergoing lobectomy, which could in part explain the association between lobectomy and better survival outcomes. However, when limited to patients with 7 or more lymph nodes examined, Cox model including propensity score as a continuous variable showed that patients undergoing lobectomy had a significantly better LCSS. HR also favored lobectomy regarding OS and LCSS in matched analysis, although statistical significance was not reached, probably due to the small number of cases (105 lobectomy and 105 segmentectomy).

A recent study by Razi and colleagues also used SEER database. They found that in elderly patients (75 years or older) with T1aN0M0 NSCLC, sublobar resection is not inferior to lobectomy [18]. Combining their results with our findings, we could conclude that either elder age (75 years or older) or small tumor size (2 cm or less) alone could not justify the application of segmentectomy. However, segmentectomy might be a reasonable alternative in elderly patients (75 years or older) with T1aN0M0 NSCLC. Yendamuri and colleagues [19] also investigated SEER database, and found that the survival benefit of lobectomy over sublobar resection decreased over the past 2 decades. That is the reason why we adjusted year of diagnosis in our study.

In conclusion, our study showed that elderly (≥ 70 years) patients with stage I ≤ 3 cm NSCLC treated with segmentectomy had significantly worse OS and LCSS compared to those undergoing lobectomy. Elder age alone could not justify the application of segmentectomy in early-stage lung cancer. Prospective randomized trials are warranted to determine the optimal surgical resection for elderly patients.

## MATERIALS AND METHODS

This retrospective study used data from the publically available SEER database (1988-2012, data submitted in November 2014) through on-line access with the SEER*Stat software version 8.2.1-alpha. The Institutional Review Board of Fudan University Shanghai Cancer Center approved this study.

Patients eligible for this study should meet the following criteria: (1) lung cancer as the first primary malignancy; (2) histology codes denoting non-small cell lung cancer; (3) age at diagnosis ≥ 70 years old; (4) underwent lobectomy or segmentectomy; (5) patients were diagnosed from 1998 to 2012 as SEER codes for segmentectomy was not available until 1998; (6) stage I and tumor size ≤ 30 mm. Patients without sufficient information on pathologic stage or tumor size were excluded. Patients who received radiation therapy prior to surgery were excluded.

We collected the following data for individual patient: age at diagnosis, sex, race, marital status, year of diagnosis, type of surgery, number of lymph nodes examined, pathologic stage, tumor size, tumor histology, tumor grade, information of radiation therapy, lung cancer-specific survival (LCSS) and overall survival (OS).

### Statistical analysis

Pearson's chi-squared test or Fisher's exact test was used to compare correlation between type of surgery and a categorical variable. Comparison of differences of a continuous variable between patients who received lobectomy and segmentectomy was performed using independent sample *t* test. Propensity score matching was used to adjust for the potential difference in the baseline characteristics between patients undergoing different surgical approaches. Logistic regression including age (treated as a continuous variable), gender, race, marital status, year of diagnosis, tumor size (treated as a continuous variable), tumor histology and grade was used to estimate propensity scores.

Cox regression multivariate survival analysis adjusting for propensity scores was conducted to compare the OS and LCSS of patients undergoing segmentectomy or lobectomy. Hazard ratio (HR) and its 95% confidence interval (CI) were calculated. First, the propensity score was included as a continuous variable in a Cox model. Second, Cox model was calculated within categories defined by propensity score quintiles. Third, patients receiving segmentectomy and lobectomy were matched (1:1) according to their propensity scores, and Cox model was performed to compare survival [3]. Subgroup analysis was conducted in patients with tumors ≤ 2 cm in size, patients ≥ 75 years old and patients with 7 or more lymph nodes examined. Cox analysis within strata defined by propensity score quintiles was not performed in subgroup analysis because of limited number of cases.

Statistical analysis was done in Stata (version SE/11, StataCorp, Texas). All tests were two-tailed, and *P* < 0.05 was considered as statistically significant.
